# Allelic diversity and patterns of selection at the major histocompatibility complex class I and II loci in a threatened shorebird, the Snowy Plover (*Charadrius nivosus*)

**DOI:** 10.1186/s12862-020-01676-7

**Published:** 2020-09-10

**Authors:** Medardo Cruz-López, Guillermo Fernández, Helen Hipperson, Eduardo Palacios, John Cavitt, Daniel Galindo-Espinosa, Salvador Gómez del Angel, Raya Pruner, Oscar Gonzalez, Terry Burke, Clemens Küpper

**Affiliations:** 1 Posgrado en Ciencias del Mar y Limnología, Universidad Nacional Autónoma de México, Ciudad Universitaria, 04510 Cd. México, Mexico; 2 Unidad Académica Mazatlán, Instituto de Ciencias del Mar y Limnología, Universidad Nacional Autónoma de México, Apartado Postal 811, 82040 Mazatlán, Sinaloa Mexico; 3 NERC Biomolecular Analysis Facility, Department of Animal and Plant Sciences, University of Sheffield, Sheffield, S10 2TN UK; 4 Centro de Investigación Científica y de Educación Superior de Ensenada, Unidad La Paz, Miraflores 334, Col. Bellavista, 23050 La Paz, Baja California Sur Mexico; 5 Avian Ecology Laboratory Department of Zoology, Weber State University, Ogden, UT 84408 USA; 6 Departamento Académico de Ciencias Marinas y Costeras, Universidad Autónoma de Baja California Sur, Carretera al Sur km 5.5, A.P. 19-B, 23080 La Paz, B.C.S. Mexico; 7 Florida Fish and Wildlife Conservation Commission, Fish and Wildlife Research Institute, Panama City, FL USA; 8 Grupo Aves del Perú, Gómez del Carpio 135, Barrio Medico, 34 Lima, Peru; 9 Department of Natural Sciences, Emmanuel College, Franklin Springs, GA 30369 USA; 10 Max Planck Institute for Ornithology, Eberhard-Gwinner-Strasse, 82319 Seewiesen, Germany

**Keywords:** Major histocompatibility complex (MHC), Balancing selection, Peptide-binding region (PBR), MHC class I, MHC class II, Private alleles, *Charadrius*

## Abstract

**Background:**

Understanding the structure and variability of adaptive loci such as the major histocompatibility complex (MHC) genes is a primary research goal for evolutionary and conservation genetics. Typically, classical MHC genes show high polymorphism and are under strong balancing selection, as their products trigger the adaptive immune response in vertebrates. Here, we assess the allelic diversity and patterns of selection for MHC class I and class II loci in a threatened shorebird with highly flexible mating and parental care behaviour, the Snowy Plover (*Charadrius nivosus*) across its broad geographic range.

**Results:**

We determined the allelic and nucleotide diversity for MHC class I and class II genes using samples of 250 individuals from eight breeding population of Snowy Plovers. We found 40 alleles at MHC class I and six alleles at MHC class II, with individuals carrying two to seven different alleles (mean 3.70) at MHC class I and up to two alleles (mean 1.45) at MHC class II. Diversity was higher in the peptide-binding region, which suggests balancing selection. The MHC class I locus showed stronger signatures of both positive and negative selection than the MHC class II locus. Most alleles were present in more than one population. If present, private alleles generally occurred at very low frequencies in each population, except for the private alleles of MHC class I in one island population (Puerto Rico, lineage *tenuirostris*).

**Conclusion:**

Snowy Plovers exhibited an intermediate level of diversity at the MHC, similar to that reported in other Charadriiformes. The differences found in the patterns of selection between the class I and II loci are consistent with the hypothesis that different mechanisms shape the sequence evolution of MHC class I and class II genes. The rarity of private alleles across populations is consistent with high natal and breeding dispersal and the low genetic structure previously observed at neutral genetic markers in this species.

## Background

The genes of the major histocompatibility complex (MHC) are crucial for the immune response in vertebrates [1, 2]. Their encoded proteins are involved in presenting antigen derived from pathogens to immune cells, which then trigger a cascade of immune responses [3, 4]. Because of their functional importance and the direct link between MHC diversity, fitness and individual behaviour [3, 5], the MHC has been the subject of ecological and evolutionary studies ranging from assessing individual survival and mate choice to the processes of speciation and practical conservation management [6–10]. Adaptive genes, which are directly associated with individual fitness, are important for population viability and hence conservation [2, 11]. The loss of adaptive genetic diversity has been associated with an increase in the risk of extinction, especially in species with low population sizes [11]. The maintenance of MHC diversity is crucial for pathogen resistance, which represents one of the principal selective forces impacting fitness and long-term survival of endangered species [2, 12].

MHC genes display the highest degree of polymorphism within vertebrate genomes [5, 13]. Pathogen-mediated selection results in positive selection and the substitution of amino acids in the codons of the peptide-binding region (PBR), as well as balancing selection including heterozygote advantage, frequency-dependent selection and fluctuating selection [2, 4, 14]. Recently, a number of studies showed that the evolutionary dynamics of the MHC genes is driven by high rates of recombination, duplication and conversion [15–17]. Through these processes populations can respond to a great number of antigens [10, 17]. The MHC genes are divided into two principal classes: class I, which is responsible for immune defence against intracellular pathogens such as viruses, and class II, which is responsible for dealing with extracellular pathogens such as bacteria and nematodes [2].

The number of MHC genes varies between and within species [10]. In mammals, MHC genes are organized into a dense genomic region and are characterized by complex organization and many pseudogenes, leading to extraordinary genetic diversity. For example, in humans approximately 9000 class I alleles and 3000 class II alleles have been described [18]. In birds, the structure and organization of the MHC region varies not only between, but also within the same family [9]. Some groups, such as chicken *Gallus gallus* and some birds of prey, have an extraordinarily compact MHC region (coined as the “minimal essential” MHC, [19, 20]). However, other galliform species have duplications, leading to many MHC alleles [21, 22]. In contrast, in Passeriformes, the MHC shows a complex architecture, and is frequently composed of multiple expressed loci and pseudogenes [1, 23, 24]. Other groups of birds, such as the Charadriiformes, appear to have a diversity and complexity intermediate between chicken and Passeriformes [25, 26]. The differences in the number and organization of the MHC genes in vertebrates might be best explained by different evolutionary dynamics in the birth and death of genes [27]. Here, new genes are generated by duplication, with some daughter copies conserving their function while others are inactivated or eliminated from the population [10, 27].

Within the order Charadriiformes, study of the MHC have so far been restricted to three families: Alcidae, Laridae and Scolopacidae. These studies revealed considerable differences in the diversity and organization of the Charadriiform MHC [16, 25, 26, 28, 29]. To investigate which mechanisms generate and promote MHC evolution and diversity [9], studies in more phylogenetically distinct families are required.

We investigated the diversity and organization of the MHC in the Snowy Plover *Charadrius nivosus*, a member of the Charadriidae [30]. Until recently, Snowy Plovers were lumped with Kentish Plovers *Charadrius alexandrinus* and considered to be a cosmopolitan species, but the analysis of genetic and phenotypic traits has shown that the two species are separate and that Snowy Plovers are characterized by low genetic diversity at neutral genetic markers [31–33]. Three Snowy Plover lineages are commonly recognized and distinguishable with genetic markers: Western Snowy Plovers (*C. n. nivosus*) in North America, Cuban Snowy Plovers (*C. n. tenuirostris*) in the Caribbean, and the Peruvian Snowy Plover (*C. n. occidentalis*) in South America [31]. Coastal populations in particular have declined throughout the range and now require active conservation management [34–37]. Here, we develop markers for MHC class I and class II genes to examine adaptive genetic diversity in Snowy Plovers. Our study has four aims: First, we characterize functionally essential regions of the MHC class I and class II loci to provide novel genetic markers for studying adaptive diversity in this species. Second, we investigate the genetic diversity (number of segregating sites and nucleotide diversity), evolutionary distance and type of selection acting on MHC class I and class II alleles. Third, we compare the diversity of MHC classes I and II with the respective diversity observed in other Charadriiformes. Finally, we describe the pattern of diversity across the geographic range and test for the presence of private alleles within Snowy Plover populations.

## Results

### MHC class I: exon 3

**Fig. 1:**
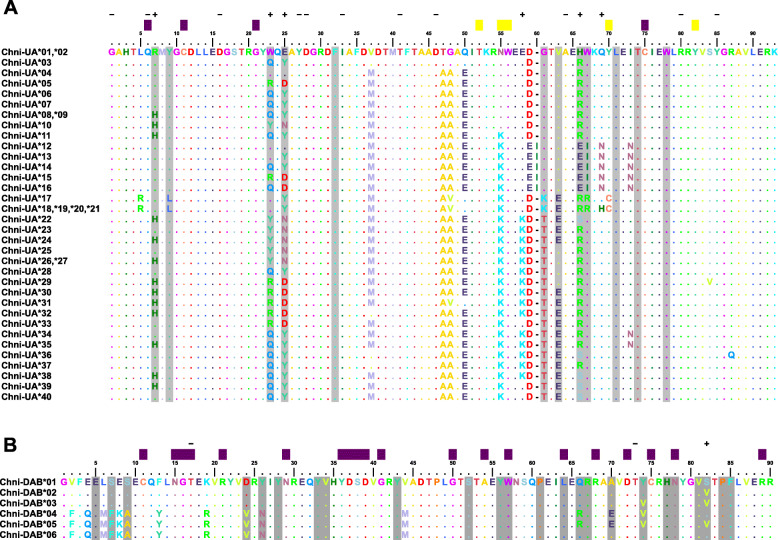
Alignments of MHC class I alleles (**a**) and MHC class II alleles (**b**) detected among sampled Snowy Plovers *Charadrius nivosus*. Alleles with an identical translation of amino acids are shown in a single sequence. Dots indicate identity to the consensus sequence and dashes indicate gaps. Gray boxes indicate the peptide binding regions (PBR) inferred for MHC class I and MHC class II. Intra- and inter-domain contacts are in purple, and main chain binding sites to peptides are in yellow. Sites under positive (+) and negative (−) selection were identified using FUBAR (Murrel et al. 2013; http://www.datamonkey.org/)

We discarded 32 samples because they did not pass the quality filters or because they did not have the minimum number of reads per amplicon. Among the 218 remaining samples from eight populations, we found a total of 40 alleles. Five alleles (‘long alleles’; Chni-UA*12 to 16) showed a 3-bp insertion at position 178–180 (amino acid position 60 in the alignment) in the α2 domain, retaining the correct reading frame. Meanwhile, the remaining 35 alleles (‘short alleles’; Chni-UA*01 to UA*11 and Chni-UA*17 to UA*40) did not show this insertion. The alignment of exon 3 displayed five of the eight highly conserved amino acid (peptide main-chain) sites in birds (amino acids: TKWYY, Fig. 1). Alleles Chni-UA*01 to UA*10 had an amino acid substitution at the second of these conserved sites (N for K), whereas in alleles Chni-UA*17 to UA*21 the fourth site was substituted (C for Y). Four of the 18 highly conserved intra- and interdomain contacts described in vertebrates [38] were present, and none of these showed a polymorphism.

#### Allelic diversity

We found two to seven alleles per individual ($\overline{\mathrm{x}}$ ± SD: 3.70 ± 0.92), which suggested that we obtained alleles from up to four loci, assuming heterozygosity. We detected up to three non-classical alleles in 216 of 218 samples. The number of alleles did not differ between individuals across populations (Table S1). However, we found differences in the number of alleles per population, with populations Nayarit and Puerto Rico showing fewer alleles than the other populations (Table S2). Long alleles were less common among the 218 individuals genotyped, with only 23 individuals displaying one or two long alleles, whereas the short alleles were present in all individuals. Chni-UA*21 was the most common allele and detected in 173 individuals (83.5%), followed by Chni-UA*20 in 102 individuals (49.2%) and Chni-UA*30 in 69 individuals (33.3%). All individuals genotyped had at least two alleles. Most individuals (48.3%) had four alleles, 24.6% had three alleles, 13% had five alleles, 11.6% had two alleles, 2.4% had six alleles and 0.9% had seven alleles.

#### Diversity and inference of selection

The average nucleotide diversity (π) for the complete sequence was similar among the three lineages, ranging from 0.05 to 0.07. Populations at Nayarit and Puerto Rico showed the highest levels of nucleotide diversity, the nucleotides distance and average nucleotide diversity at PBR sites, whereas populations at Utah, San Quintin and Ceuta had the lowest levels of nucleotide diversity (both complete sequence and PBR sites) and nucleotide distance (Table 1). Within all populations, PBR sites showed higher diversity than non-PBR sites, suggesting balancing selection at these sites (Table 1). Bayesian analysis of selection (FUBAR: Fast Unconstrained Bayesian AppRoximation) identified six sites (sites 7, 23, 25, 58, 66 and 69) under positive selection and 11 sites (sites 1, 6, 16, 27, 28, 33, 41, 46, 64, 80 and 85) that displayed diversity patterns consistent with negative, purifying selection (Fig. 1a). Sites with purifying selection were exclusively located in the non-PBR region. Also, differences in nonsynonymous substitution rate and synonymous substitution rate (*dN*/*dS*) suggested stronger positive selection at PBR sites in comparison with non-PBR sites (Table 1). Among genetic lineages, *C. n. tenuirostris* (Puerto Rican population) showed the lowest level of positive selection. Using GARD (Genetic Algorithm for Recombination Detection) we found no evidence for recombination among the 40 alleles.

**Table 1:** Diversity at MHC class I exon 3 in the Snowy Plover (*Charadrius nivosus*). Segregating sites of amino acids (*S*_aa_), average nucleotide diversity (π), evolutionary distance of nucleotide sequences (*d*_nt_) and amino acid sequences (*d*_aa_). The measures of diversity, and the synonymous and non-synonymous substitution rates, were calculated for the complete sequence (All), and separately for the PBR and non-PBR sites within each population. The number of genotyped individuals per population (*N*) and the number of samples that passed the quality filters (*n*) are shown. To have comparable measures of diversity we randomly selected 15 allele sequences for each population, with the exception of Nayarit and Puerto Rico, where we found only 13 and 9 alleles, respectively

Population	Lat	Long	*N* (*n*)		*dN* ± S.E	*dS* ± S.E	*dN* /*dS*	*S*_aa_	π	*d*_nt_ ± S.E	*d*_aa_ ± S.E
*C. n. nivosus* (UTA)	40.9	−112.13	40 (40)	All	0.05 ± 0.01	0.08 ± 0.02	0.62	21	0.06	0.07 ± 0.01	0.09 ± 0.02
				PBR	0.33 ± 0.13	0.12 ± 0.07	2.75	8	0.21	0.35 ± 0.15	0.34 ± 0.09
				Non-PBR	0.02 ± 0.01	0.08 ± 0.02	0.25	13	0.04	0.04 ± 0.01	0.05 ± 0.01
*C. n. nivosus* (SAQ)	30.4	− 115.97	47 (35)	All	0.05 ± 0.01	0.08 ± 0.02	0.62	20	0.05	0.06 ± 0.01	0.08 ± 0.02
				PBR	0.34 ± 0.12	0.11 ± 0.07	3.10	8	0.21	0.36 ± 0.21	0.34 ± 0.08
				Non-PBR	0.02 ± 0.01	0.07 ± 0.02	0.28	12	0.03	0.03 ± 0.01	0.04 ± 0.01
*C. n. nivosus* (CEU)	23.91	− 106.95	49 (49)	All	0.05 ± 0.01	0.07 ± 0.02	0.71	20	0.05	0.06 ± 0.01	0.08 ± 0.02
				PBR	0.33 ± 0.12	0.11 ± 0.07	3.00	8	0.21	0.35 ± 0.16	0.35 ± 0.08
				Non-PBR	0.02 ± 0.01	0.07 ± 0.02	0.28	12	0.03	0.03 ± 0.01	0.04 ± 0.01
*C. n. nivosus* (NAY)	22.41	−105.62	13 (10)	All	0.06 ± 0.01	0.13 ± 0.03	0.46	19	0.07	0.07 ± 0.01	0.09 ± 0.02
				PBR	0.38 ± 0.14	0.18 ± 0.11	2.11	8	0.25	0.46 ± 0.29	0.37 ± 0.08
				Non-PBR	0.02 ± 0.01	0.12 ± 0.03	0.17	11	0.05	0.05 ± 0.01	0.05 ± 0.02
*C. n. nivosus* (TEX)	19.46	−98.97	23 (14)	All	0.06 ± 0.01	0.09 ± 0.02	0.67	19	0.06	0.07 ± 0.01	0.09 ± 0.02
				PBR	0.39 ± 0.14	0.14 ± 0.09	2.80	8	0.23	0.42 ± 0.21	0.37 ± 0.08
				Non-PBR	0.02 ± 0.01	0.09 ± 0.02	0.22	11	0.03	0.04 ± 0.01	0.05 ± 0.01
*C. n. nivosus* (FLO)	30.02	−85.57	40 (36)	All	0.05 ± 0.01	0.10 ± 0.02	0.50	19	0.06	0.07 ± 0.01	0.08 ± 0.02
				PBR	0.35 ± 0.12	0.13 ± 0.08	2.70	8	0.22	0.38 ± 0.19	0.36 ± 0.08
				Non-PBR	0.02 ± 0.01	0.09 ± 0.02	0.22	11	0.04	0.04 ± 0.01	0.05 ± 0.01
*C. n. occidentalis* (PER)	−13.84	−76.24	21 (21)	All	0.06 ± 0.01	0.09 ± 0.02	0.70	20	0.06	0.07 ± 0.01	0.09 ± 0.02
				PBR	0.36 ± 0.13	0.14 ± 0.08	2.60	8	0.23	0.41 ± 0.19	0.37 ± 0.09
				Non-PBR	0.02 ± 0.01	0.09 ± 0.02	0.22	12	0.03	0.04 ± 0.01	0.05 ± 0.01
*C. n. tenuirostris* (PUR)	17.93	−67.18	17 (13)	All	0.06 ± 0.01	0.15 ± 0.04	0.40	18	0.07	0.07 ± 0.01	0.09 ± 0.02
				PBR	0.36 ± 0.15	0.22 ± 0.13	1.64	8	0.24	0.49 ± 0.36	0.34 ± 0.07
				Non-PBR	0.03 ± 0.01	0.14 ± 0.04	0.21	10	0.05	0.05 ± 0.01	0.06 ± 0.02

#### Comparison and phylogenetic relationships with other Charadriiformes

Average nucleotide diversity and number of segregation sites were lower than those reported from Red Knot, Icelandic Black-tailed Godwit and Red-billed Gull (Table 2; Figure S1). Nevertheless, the *dN*/*dS* proportion at PBR sites was higher for the Snowy Plover, indicating stronger positive selection at these sites in comparison with the other three charadriiform species.

**Table 2:** Comparison of genetic diversity at MHC class I exon 3 in four different charadriiform species. Segregating sites of amino acids (*S*_aa_), nucleotides (*S*_nt_), and average nucleotide diversity (π), from 21 sequences randomly chosen by each of these species. Diversity indices and the synonymous (*dS*) and non-synonymous (*dN*) substitution rates were calculated for the complete sequences (All), the PBR and the non-PBR sites

	*dN *± S.E	*dS* ± S.E	*dN/dS*	*S*_nt_	*S*_aa_	π
Snowy Plover (*Charadrius nivosus nivosus*)						
All	0.05 ± 0.01	0.07 ± 0.02	0.71	47	19	0.05
PBR	0.29 ± 0.10	0.09 ± 0.05	3.22	19	8	0.19
Non-PBR	0.02 ± 0.01	0.06 ± 0.02	0.33	28	11	0.03
Red-billed Gull (*Chroicocephalus scopulinus*)						
All	0.08 ± 0.01	0.09 ± 0.02	0.90	64	34	0.08
PBR	0.36 ± 0.13	0.20 ± 0.13	1.80	20	9	0.24
Non-PBR	0.05 ± 0.01	0.08 ± 0.02	0.62	44	25	0.05
Red Knot (*Calidris canutus*)						
All	0.13 ± 0.02	0.15 ± .02	0.87	110	53	0.11
PBR	0.46 ± 0.17	0.24 ± 0.11	1.92	25	9	0.28
Non-PBR	0.09 ± 0.01	0.14 ± 0.03	0.64	85	44	0.09
Black-tailed Godwit (*Limosa limosa*)						
All	0.09 ± 0.02	0.10 ± 0.02	0.90	70	35	0.09
PBR	0.36 ± 0.14	0.32 ± 0.18	1.12	23	9	0.26
Non-PBR	0.05 ± 0.01	0.08 ± 0.02	0.62	47	26	0.05

**Fig. 2:**
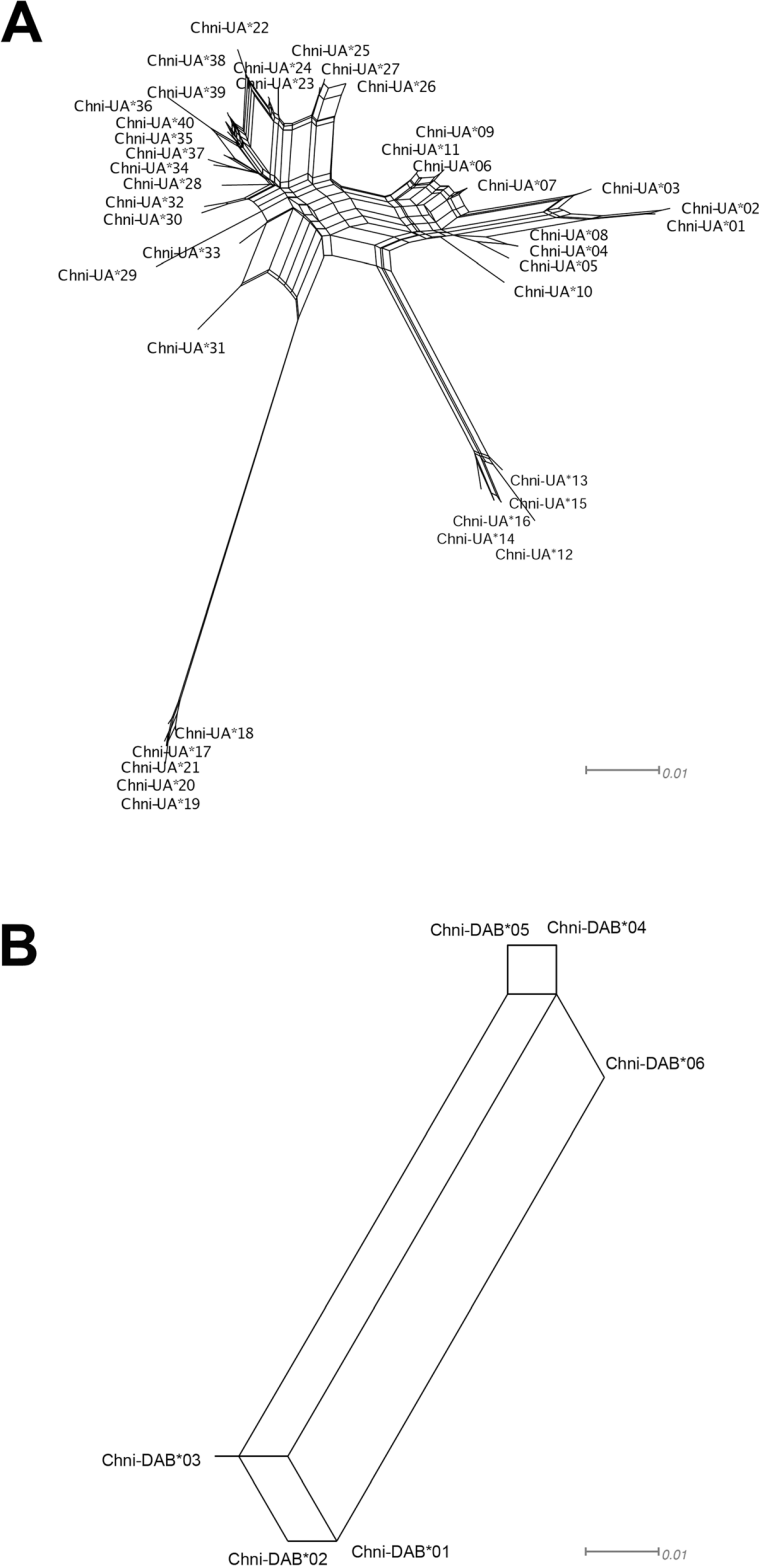
Phylogenetic network showing relationships among Snowy Plover MHC class I alleles (**a**), and MHC class II alleles (**b**) characterized from eight populations. Long alleles at MHC class I are represented with a white square, whereas non-classical alleles are represented by black squares

**Fig. 3:**
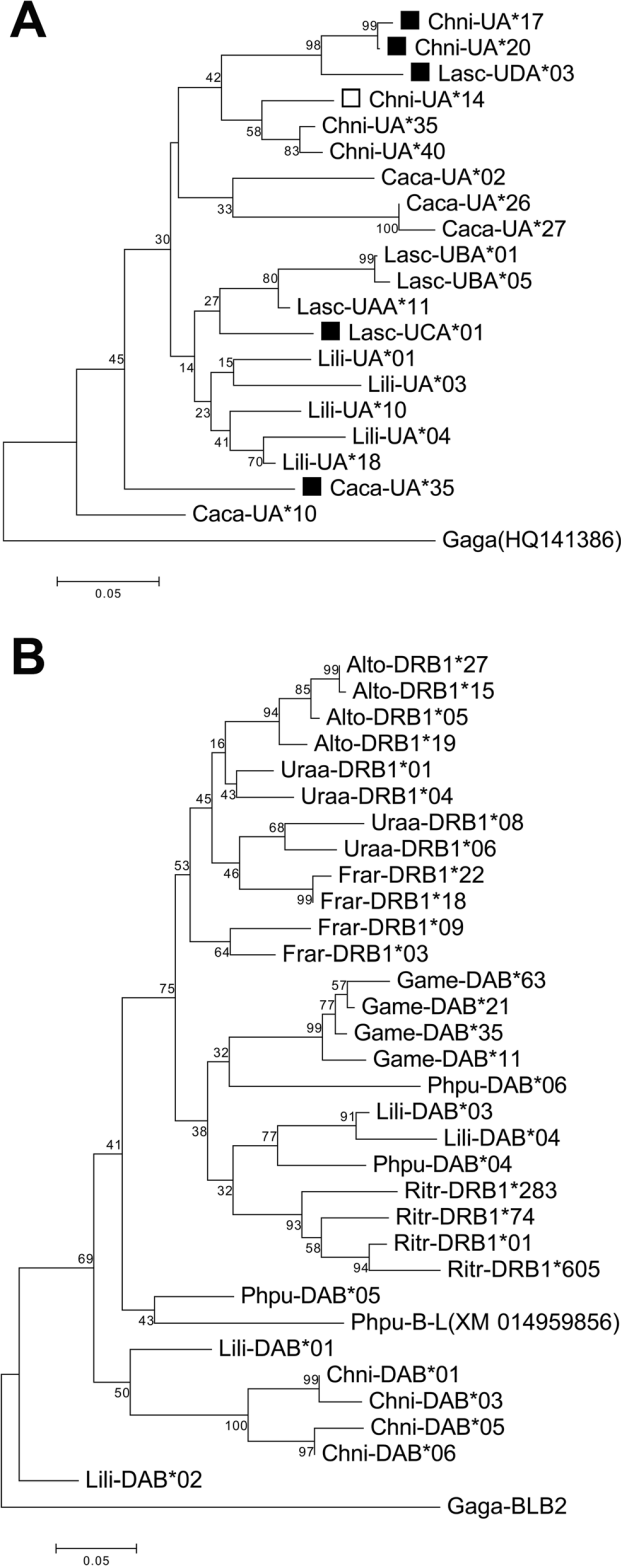
Neighbour-joining tree of amino acid sequences for MHC class I and II from different Charadriiformes. For MHC class I, four species are represented (Snowy Plover (Chni), Black-tailed Godwit (Lili), Red Knot (Caca) and Red-billed Gull (Lasc)) and (**b**) MHC class II represented by eight species (Snowy Plover (Chni), Black-tailed Godwit (Lili), Ruff (Phpu), Great Snipe (Game), Black-legged Kittiwake (Ritr), Razorbill (Alto), Common Murre (Uraa) and Atlantic Puffin (Frar)). Long alleles are indicated by a white square, non-classical alleles with a black square. The homologous MHC sequences of the chicken *Gallus gallus* (Gaga-HQ141386 and Gaga-BLB2) served as an outgroup

As in Red Knots and Red-billed Gulls, we found alleles with putatively non-classical functions among the Snowy Plover alleles. These non-classical alleles (Chni-UA*17 to UA*21) formed a well-defined cluster in the phylogenetic network (Fig. 2a) and showed a low level of polymorphism (Figs. 1a and 2a). Non-classical alleles were present in 216 of 218 samples. These alleles clustered together with the Red-billed Gull non-classical allele Lasc-UDA*03 in the neighbour-joining tree (Fig. 3a).

### MHC class II: exon 2

We discarded 36 samples, as they did not pass the quality filters. In total, we found six alleles across 214 individuals of eight populations. We found no more than two alleles per individual ($\overline{\mathrm{x}}$ ± SD: 1.45 ± 0.50), which suggests that we genotyped only one locus. The most common allele was Chni-DAB*01, which we detected in 171 individuals (79.9%), whereas the least common allele was Chni-DAB*03, present in four individuals (1.9%). Most individuals (55.6%) were homozygous.

#### Diversity and inference of selection

The nucleotide diversity (π) for subspecies *nivosus* and *occidentalis* were similar, with the PBR sites showing higher diversity in comparison to the non-PBR sites, suggesting that balancing selection could be acting at PBR sites (Table 3). The Bayesian site-by-site test in FUBAR suggested positive selection for one site (site 82), and purifying selection for two sites (sites 17 and 73, Fig. 1b). Sites with purifying selection were exclusively located in the non-PBR region. The analysis of rates of changes *dN*/*dS* indicated positive selection for the PBR sites in comparison to non-PBR sites for the *nivosus* and *occidentalis* subspecies (Table 3). All *tenuirostris* samples were homozygous. GARD identified one recombination breakpoint, which was located at position 222. When we re-ran a codon-specific model for the non-recombinant fragment of the sequences, our results remained unchanged.

**Table 3:** Diversity at the MHC class II (segregating sites of amino acids (*S*_aa_) and average diversity of nucleotides (π)) and evolutionary distance (nucleotide sequences (*d*_nt_) and amino acids (*d*_aa_)) of the alleles of the exon 2 in the Snowy Plover (*Charadrius nivosus*). The measures of diversity and the synonymous and non-synonymous substitution rates were calculated for the complete sequences (All), the PBR and the non-PBR sites for each population. The number of genotyped individuals per population (*N*) and number of samples that passed the quality filter (*n*) are shown

Population	Lat	Lon	*N* (*n*)		*dN* ± S.E	*dS* ± S.E	*dN* /*dS*	*S*_aa_	π	*d*_nt_ ± S.E	*d*_aa_ ± S.E
*C. n. nivosus* (UTA)	40.9	−112.13	40 (40)	All	0.06 ± 0.01	0.10 ± 0.03	0.60	15	0.06	0.07 ± 0.01	0.10 ± 0.02
				PBR	0.15 ± 0.05	0.08 ± 0.05	1.87	8	0.11	0.15 ± 0.05	0.20 ± 0.06
				Non-PBR	0.03 ± 0.01	0.11 ± 0.04	0.27	7	0.05	0.05 ± 0.01	0.07 ± 0.02
*C. n. nivosus* (SAQ)	30.4	−115.97	47 (36)	All	0.06 ± 0.01	0.10 ± 0.03	0.60	15	0.06	0.07 ± 0.01	0.10 ± 0.02
				PBR	0.15 ± 0.05	0.08 ± 0.05	1.87	8	0.11	0.15 ± 0.05	0.20 ± 0.06
				Non-PBR	0.03 ± 0.01	0.11 ± 0.04	0.27	7	0.05	0.05 ± 0.01	0.07 ± 0.02
*C. n. nivosus* (CEU)	23.91	−106.95	49 (34)	All	0.06 ± 0.01	0.11 ± 0.03	0.54	15	0.06	0.08 ± 0.01	0.10 ± 0.02
				PBR	0.14 ± 0.05	0.10 ± 0.05	1.40	8	0.12	0.15 ± 0.05	0.19 ± 0.05
				Non-PBR	0.03 ± 0.01	0.11 ± 0.04	0.27	7	0.05	0.05 ± 0.01	0.07 ± 0.02
*C. n. nivosus* (NAY)	22.41	−105.62	13 (10)	All	0.07 ± 0.02	0.12 ± 0.04	0.58	15	0.07	0.08 ± 0.01	0.11 ± 0.02
				PBR	0.17 ± 0.06	0.09 ± 0.05	1.88	8	0.13	0.17 ± 0.05	0.22 ± 0.06
				Non-PBR	0.04 ± 0.01	0.13 ± 0.05	0.30	7	0.05	0.06 ± 0.01	0.07 ± 0.02
*C. n. nivosus* (TEX)	19.46	−98.97	23 (17)	All	0.05 ± 0.01	0.09 ± 0.03	0.55	15	0.06	0.07 ± 0.01	0.09 ± 0.02
				PBR	0.14 ± 0.05	0.07 ± 0.05	2.00	8	0.11	0.13 ± 0.04	0.19 ± 0.05
				Non-PBR	0.03 ± 0.01	0.10 ± 0.04	0.30	7	0.04	0.04 ± 0.01	0.05 ± 0.02
*C. n. nivosus* (FLO)	30.02	−85.57	40 (39)	All	0.07 ± 0.02	0.12 ± 0.04	0.58	15	0.07	0.08 ± 0.01	0.11 ± 0.02
				PBR	0.17 ± 0.06	0.09 ± 0.06	1.88	8	0.13	0.17 ± 0.05	0.22 ± 0.06
				Non-PBR	0.04 ± 0.01	0.13 ± 0.05	0.30	7	0.05	0.06 ± 0.01	0.07 ± 0.02
*C. n. occidentalis* (PER)	−13.84	−76.24	21 (21)	All	0.07 ± 0.02	0.12 ± 0.04	0.58	15	0.07	0.08 ± 0.01	0.11 ± 0.02
				PBR	0.17 ± 0.06	0.09 ± 0.06	1.88	8	0.13	0.17 ± 0.05	0.22 ± 0.06
				Non-PBR	0.04 ± 0.01	0.13 ± 0.05	0.30	7	0.05	0.06 ± 0.01	0.07 ± 0.02
*C. n. tenuirostris* (PUR)	17.93	−67.18	17 (17)	All	–	–	–	–	–	–	–
				PBR	–	–	–	–	–	–	–
				Non-PBR	–	–	–	–	–	–	–

#### Diversity and phylogenetic relationships within the Charadriiformes

The average nucleotide diversity of Snowy Plovers (π = 0.06) was well within the range of diversity observed in other Charadriiformes (range 0.02 to 0.15, Table 4; Figure S1). This was also true for the nucleotide diversity at PBR sites (Table 4). The phylogenetic network and the neighbor joining tree showed two distant allele groups for MHC Class II (Figs. 2b and 3b). The neighbour-joining tree showed that MHC class II alleles of Snowy Plovers (Chni-DAB) were located on a different branch than most other charadriiform MHC II alleles identified so far (Fig. 3b).

**Table 4:** Comparison of genetic diversity at MHC class II (segregating sites of amino acids (*S*_aa_) and nucleotides (*S*_nt_) and average nucleotide diversity (π)) in eight species of Charadriiformes, from six alleles randomly selected for each species (except for Black-tailed Godwit and Ruff where only four alleles were available). The measures of diversity and the synonymous and non-synonymous substitution rates were calculated for the complete sequences (All), the PBR and the non-PBR sites

	*dN* ± S.E	*dS* ± S.E	*dN*/*dS*	*S*_nt_	*S*_aa_	π
Snowy Plover (*Charadrius nivosus nivosus)*						
All	0.06 ± 0.01	0.10 ± 0.03	0.60	31	15	0.06
PBR	0.14 ± 0.05	0.10 ± 0.05	1.40	15	8	0.12
Non-PBR	0.03 ± 0.01	0.11 ± 0.04	0.27	16	7	0.05
Common Murre (*Uria aalge)*						
All	0.09 ± 0.02	0.05 ± 0.02	1.80	42	26	0.07
PBR	0.19 ± 0.05	0.11 ± 0.05	1.72	22	12	0.15
Non-PBR	0.06 ± 0.02	0.04 ± 0.02	1.50	20	14	0.05
Razorbill (*Alca torda*)						
All	0.03 ± 0.01	0.02 ± 0.01	1.50	17	10	0.02
PBR	0.07 ± 0.03	0.02 ± 0.02	3.50	10	6	0.05
Non-PBR	0.02 ± 0.01	0.02 ± 0.01	1.00	7	4	0.01
Atlantic Puffin (*Fratercula arctica*)						
All	0.10 ± 0.02	0.05 ± 0.02	2.00	44	26	0.08
PBR	0.26 ± 0.07	0.04 ± 0.03	6.50	24	14	0.16
Non-PBR	0.05 ± 0.01	0.06 ± 0.02	0.83	20	12	0.05
Black-legged Kittiwake (*Rissa tridactyla*)						
All	0.12 ± 0.02	0.06 ± 0.02	2.00	56	31	0.09
PBR	0.29 ± 0.06	0.05 ± 0.03	5.80	29	15	0.18
Non-PBR	0.07 ± 0.02	0.07 ± 0.02	1.00	27	16	0.06
Great Snipe (*Gallinago media*)						
All	0.03 ± 0.01	0.04 ± 0.01	0.75	20	10	0.03
PBR	0.08 ± 0.03	0.04 ± 0.03	2.00	10	6	0.06
Non-PBR	0.02 ± 0.01	0.04 ± 0.02	0.50	10	4	0.02
Ruff (*Philomachus pugnax*)						
All	0.17 ± 0.03	0.15 ± 0.04	1.13	67	36	0.14
PBR	0.33 ± 0.09	0.18 ± 0.01	1.83	29	15	0.23
Non-PBR	0.12 ± 0.02	0.14 ± 0.03	0.85	38	21	0.11
Black-tailed Godwit (*Limosa limosa*)						
All	0.18 ± 0.03	0.14 ± 0.03	1.28	68	40	0.15
PBR	0.31 ± 0.08	0.21 ± 0.08	1.47	27	16	0.23
Non-PBR	0.14 ± 0.03	0.12 ± 0.04	1.16	41	24	0.12

### Geographic pattern of MHC diversity

**Fig. 4:**
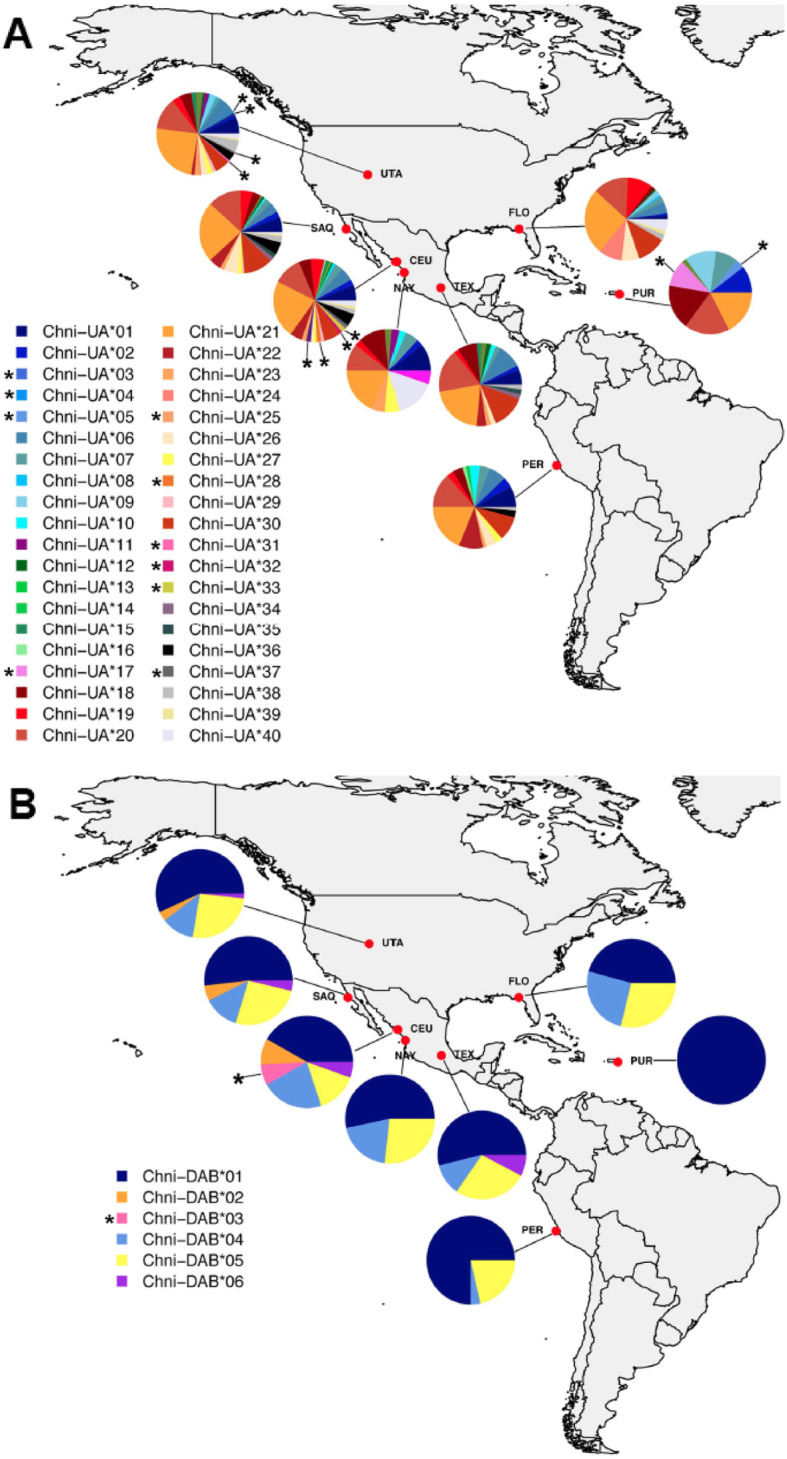
Geographical distribution of MHC class I (**a**) and II (**b**) alleles among eight sampled Snowy Plover populations. Populations are abbreviated (Utah = UTA San Quintin = SAQ, Ceuta = CEU, Nayarit = NAY, Texcoco = TEX, Peru = PER, Florida = FLO and Puerto Rico = PUR). Long alleles of the MHC class I are in green colors and the asterisk shows alleles exclusive to single populations. The figure was created using software RStudio (Version 1.0.153; https://rstudio.com/)

We found that 25% of alleles were private alleles for MHC class I and 16.7% private alleles for MHC class II. For MHC class I, Snowy Plover populations from Utah, Ceuta and Puerto Rico showed private alleles (Fig. 4a). For Utah, four (Chni-UA*03, 04, 31 and 37) of 30 alleles were private, for Ceuta, four (Chni-UA*25, 28, 32 and 33) of 34 alleles were private and for Puerto Rico, two (Chni-UA*05 and 17) of nine alleles were private. Other populations lacked private alleles (notably the Peruvian population of the subspecies *occidentalis*) but showed similar numbers of alleles (San Quintin: 23 alleles, Nayarit: 13 alleles, Texcoco: 19 alleles, Florida: 17 alleles and Peru: 18 alleles). With the exception of the island population of Puerto Rico (lineage *tenuirostris*), private alleles generally occurred in low frequencies (Fig. 4). At MHC class II, there were no private alleles within lineages *tenuirostris* or *occidentalis*. Only the Ceuta population had a private allele (Chni-DAB*03), and we found all six alleles in this population. The other populations had three to five alleles, except for Puerto Rico, where we detected only one allele (Fig. 4b).

## Discussion

In this study, we developed new MHC markers that amplified with high success exon 3 of MHC class I and exon 2 of MHC class II in the Snowy Plover, a shorebird species of high conservation concern. This allowed us to examine adaptive genetic diversity at this important locus in the family Charadriidae and provides novel and useful markers for future studies in other *Charadrius* species. Surveying both MHC classes in more than 200 individuals from eight populations, we report differences in allelic diversity across both MHC classes, with nearly seven times as many alleles at MHC class I than at MHC class II. The genotypic variation in individuals suggests that the markers amplified four highly similar loci for MHC class I, as we registered up to seven alleles at MHC class I, whereas a maximum of two alleles per individual is consistent with amplification of a single locus at MHC class II.

Our network analysis suggests the presence of classical and non-classical genes among the amplified MHC class I loci. Non-classical loci have evolved from classic MHC genes to perform specific tasks in the immune recognition [39]. Five alleles (Chni-UA*17 to 21), formed a well-defined cluster in the phylogenetic network, which is characteristic for non-classical alleles. Non-classical alleles have also previously been described in other shorebirds (Red-billed Gull: [29]; Red Knot: [25]). Consistent with expectations derived from other non-classical alleles, these five alleles showed a low level of polymorphism [40, 41]. Four alleles were only differentiated by synonymous substitutions, whereas the fifth allele, found only in the Puerto Rico population, deviated in a single amino acid substitution. We observed that two of three non-classical alleles Lasc-UCA*01 and Caca-UA*35 clustered with the classical alleles of their respective species Red-billed Gull and Red Knot. This suggests that they may have evolved through recent duplications that have occurred after the divergence of the species in this tree. By contrast, Lasc-UDA*03 and the Snowy Plover non-classical alleles may have been created through a more ancient duplication event.

We observed a second well-defined cluster in our phylogenetic network at MHC class I; this cluster corresponds to five alleles (Chni-UA *12 to 16) that shared a 3-bp insertion. The observed insertion kept the reading frame intact, presumably preserving their function. Contrary to the non-classical alleles, these five alleles with a 3-bp insertion show a higher nucleotide diversity, including several non-synonymous changes between these five alleles, suggesting these alleles have classical functions. In birds, insertions are less frequent than deletions in MHC genes, and it has been suggested that insertions have a reduced adaptive advantage [42]. However, some insertions may contribute to adaptive MHC variation due to possible changes in the PBR [42, 43]. Unlike the MHC class I, the six alleles at MHC class II formed an undifferentiated cluster, with both synonymous and non-synonymous substitutions present. Together, with the observation of no more than two alleles per individual, we concluded that our markers amplified a single classical MHC II locus. When we compared the nucleotide diversity at the PBR sites, we found that the MHC class I showed moderately higher values of diversity (0.16 ± 0.03) than at MHC class II (0.12 ± 0.03). This result may be a consequence of having amplified four loci at MHC I and only one locus at MHC II.

For both MHC classes, we observed that the number of sites under negative (purifying) selection was higher than sites under positive (diversifying) selection when we considered the full sequence. As PBR sites are interacting directly with the antigens derived from pathogens, it is expected that these sites are subject to stronger positive diversifying selection than non-PBR sites. Consistent with this, we found that sites with signatures of purifying selection were predominantly non-PBR sites, whereas five of the six sites under diversifying selection in the MHC class I and in one site at MHC class II corresponded to PBR sites. Similar patterns were observed in another shorebird, the Icelandic Black-tailed Godwit [26], and shown in a recent comparative study of selection at the avian MHC [42].

Our finding that more sites showed signatures of positive selection at MHC class I than at MHC class II differs from a recent global analysis of selection at the avian MHC [42]. Here, the authors suggested that the pressure of extracellular pathogens is higher in non-passerines, resulting in a stronger signature of selection for the MHC class II in non-passerines than in passerines. The order Charadriiformes does not seem to fit to this proposed rule. Consistent with Minias et al. [42], gulls and most of the alcids show a strong signature of selection at MHC class II and weaker selection at MHC class I, but the paraphyletic group of shorebirds (plovers and sandpipers) instead shows a pattern more similar to passerines [16, 25, 26, 28, 29, 44]. Several morphological or ecological variables may explain this discrepancy. First, body size may be related to parasite abundance, as larger hosts may provide a greater variety of niches and, in turn, support a higher number of parasites than smaller birds [45]. Snowy Plovers are relatively small birds with a mean body mass of 38–50 g [32, 46]. Second, the selective pressures imposed by parasites may be habitat dependent. Although aquatic birds (mainly non-passerines) show a more diverse parasite community than their terrestrial counterparts [45], there is a difference between freshwater and saltwater habitats. Snowy Plovers inhabit the shores of alkaline water bodies, such as salt lakes, salt evaporation ponds and sandy beaches [46]. These saline habitats are typically considered to have a lower abundance of extracellular parasites [47–49], which would be consistent with the observed low diversity at MHC class II. In general, shorebirds show a low prevalence in intracellular pathogens, although viral infections (West Nile virus; [50], Newcastle disease virus [50], avian influenza; [51]), avian haemosporidians (*Plasmodium* and *Haemoproteus* spp.; [52]) and bacterial infections (*Mycobacterium*; [53]) have all been reported. Furthermore, Snowy Plovers inhabit low latitudes, where the diversity of intracellular pathogens, as well as of their vectors, is expected to be high [48, 52]. In addition to the copy number variation for the fragment amplified at MHC I, the high abundance of intracellular pathogens in the tropics may contribute to the high allelic diversity at MHC class I. Further research is needed to determine whether Charadriiformes themselves show unusual group-specific variation in intra- and extracellular pathogens, or whether other life-history, ecological and evolutionary differences explain the observed differences in signatures of selection.

Across continental populations (subspecies *nivosus* and *occidentalis*), we found a similar number of alleles and nucleotide diversity. This result is consistent with the high gene flow observed among Snowy Plover populations [31, 33, 54]. At MHC I only, we found that the Nayarit population had a lower number of alleles (13 alleles), although this may reflect the sample size, as we only genotyped ten birds from this population. Among continental populations, private alleles were generally rare, and when present, occurred at low frequencies, suggesting that these represent rare alleles rather than geographically distinct alleles. By contrast, the island population of Puerto Rico (lineage *tenuirostris*) showed two privates alleles at MHC class I at moderate frequencies (Fig. 4): these alleles may represent true geographic variants. Interestingly, the *tenuirostris* population was monomorphic at MHC class II. Together with the lower positive selection at PBR sites at MHC class I, this might suggest that pathogen pressure is weaker in this population. The low genetic diversity may be a common feature of island populations that are thought to be exposed to fewer pathogens than continental pathogens [55]. For example, among shorebirds, Icelandic Black-tailed Godwits did not show positive selection at MHC class I PBR sites [26]. Other biogeographic features could contribute to the observed differences in genetic diversity. In contrast to most other populations, *tenuirostris* inhabits the Atlantic Ocean and the diversity of pathogens is thought to be lower in the Atlantic Ocean than the Pacific Ocean [56]. However, the other Atlantic population located in Florida did not show lower diversity than Pacific populations. Demography may also have played a role in shaping MHC diversity. A recent study showed that all Snowy Plover lineages went through a bottleneck within the last 1000 years, with particularly strong effects observed in *C. n. tenuirostris* [33]. A similar pattern of loss of adaptive diversity has been observed in other bird populations subject to recent bottlenecks (see [57–59]).

Comparing allelic diversity across species, we found that diversity at MHC class I in Snowy Plovers (40 alleles across four loci amplified) was similar to other Charadriiformes species (Red-billed Gull [29]; (38 alleles), Red Knot [25]; (36 alleles) and Black-tailed Godwit [26]; (47 alleles)). By contrast, MHC class II (six alleles) showed a lower allelic diversity than other Charadriiformes (Great Snipe – 50 alleles, [28]; Marbled Murrelet *Brachyramphus marmoratus* – 27 alleles, [60]). Despite the copy number variation, the nucleotide diversity at MHC class I in Snowy Plovers was lower than in other Charadriiformes (Table 2). However, the observed values were within the range of other non-passerines (such as the Humboldt Penguin *Spheniscus humboldti* – 0.03 to 0.04 – and Magellanic Penguin *Spheniscus magellanicus* – 0.05 to 0.06, [61]; or grouse species – on average 0.05, [58]). For MHC class II, we found intermediate nucleotide diversity in comparison to other Charadriiformes (Marbled Murrelet – 0.08; [60]) and other non-passerines (Magellanic Penguin – 0.02, [61]; Eurasian Coot *Fulica atra* – 0.11, [62]; and Black Grouse *Tetrao tetrix* – 0.11, [63]).

## Conclusions

We developed novel MHC markers to amplify the PBR exon 3 of MHC class I and PBR exon two of MHC class II for the threatened Snowy Plover. These are the first markers for MHC in the family Charadriidae and we anticipate that they will be of high utility for studying MHC in other plover species. Overall, genetic diversity at MHC in Snowy Plovers was low to moderate and likely to be shaped by past demographic processes such as bottlenecks and island colonization. In line with population genetic studies, we find that there is limited genetic differentiation attributable to geographic variation, consistent with the high gene flow observed in this species. Contrasting differences in the allelic diversity between MHC class I and class II indicate stronger positive selection at MHC class I than at MHC class II. These differences may reflect variation in exposure to intracellular and extracellular pathogens [42], but further studies are needed to confirm this.

## Methods

### Population sampling

We collected blood samples of 250 adult and juvenile Snowy Plovers from eight populations across North, Central and South America between 2006 and 2016 (Table 1). We captured adults in funnel traps placed on nests during incubation or used mist nets at other times using the methods described in Székely et al. [64]. After capturing and banding the individuals, we took 20 to 75 μl blood with heparinized capillaries from the brachial vein. We captured chicks at or near to the nest a few hours after hatching and took ~ 20 to 50 μl blood from the tarsal vein. We stored the blood in 1 ml of Queen’s lysis buffer [65] at 4 °C or pure ethanol at room temperature until further processing.

### DNA extraction

We isolated genomic DNA using the ammonium acetate precipitation method [66]. We checked the integrity of the DNA using a 0.8% agarose gel stained with SYBRsafe (Invitrogen). We measured DNA concentration using either a fluorometer (FLUOstar OPTIMA) or Nanodrop ND800 (Thermo Fisher Scientific).

### Primer design for the MHC loci

We designed primers to capture the most polymorphic PBR sites in exonic regions for both MHC class I and MHC class II genes in Snowy Plovers. For MHC class I, we initially used the primers MHCI-int2F [20] and MHCI-ex3R [67] to isolate exon 3 of non-passerine birds. We undertook polymerase chain reactions (PCRs) in a total volume of 20 μl containing 12 μl Multiplex PCR Master Mix (MM, Qiagen), 4 μl Q-Solution (Qiagen), and 1 μl of each primer (1 μM) and 2 μl DNA (~ 15 ng/μl). The PCR program started with an initial denaturation step at 95 °C for 15 min, followed by 30 cycles at 94 °C for 30 s, 56 °C for 90 s and 72 °C for 90 s, and a final elongation step at 72 °C for 10 min. For MHC class II, we first used primers MHC05 [68] and 305 [69], and the primers 306 [69] and RapEx3CR [70] to capture introns 1 and 2, and parts of exons 1 and 3, respectively. We ran PCRs in a total volume of 20 μl, containing 16 μl MM, 1 μl of each primer (1 μM) and 2 μl of DNA (~ 15 ng/μl). The PCR program consisted of one cycle at 95 °C for 3 min, followed by 30 cycles at 94 °C for 30 s, 60 °C for 90 s and 72 °C for 90 s, followed by a final step at 72 °C for 10 min. All PCRs were run on a thermocycler PTC-225 DNA Tetrad Engine. We visualized PCR products using an agarose gel at 1.5% stained with ethidium bromide. For MHC class II we obtained multiple bands and subsequently cut out the visible bands of the expected size and extracted the amplified fragment using the QIAquick Gel Extraction Kit (QIAGEN). For MHC class I we only observed a single band, and the product did not require gel excision. We cleaned up MHC class I and II amplicons with ExoSap and sequenced the products using the BigDye terminator v.3.1 chemistry (Applied Biosystems) on an ABI 3730 automated sequencer (Applied Biosystems). For each MHC locus we aligned the sequences of six individuals using CodonCode Aligner 5.0.2 (CodonCode Corporation). We confirmed the identity of the sequences through blast hits in GenBank (NCBI). We then designed new primers Chni-Ex2F 5′-GAACTGCCTCCCTGCACAAA-3′ and ChCR-Ex2R 5′-TTCCCCGGGGAAATGTTCT-3′ to amplify the complete exon 2 for the MHC class II; and ChCR_MHCI_Ex2aF 5′-GGGTCTGTGCCCCACT-3′ for use with primer MHCI-ex3R 5′- CTCACCTTTCCTCTCCAG-3′ [41] to amplify the complete exon 3 of MHC class I.

### Amplification and sequencing

We adopted a two-step PCR protocol to amplify the PBRs of both MHC classes and enable multiplexing. For PCR1 we created new oligonucleotides adding the Illumina overhang sequencing adapters F 5′-TCTACACGTTCAGAGTTCTACAGTCCGACGATC-3′ and R 5′-GTGACTGGAGTTCAGACGTGTGCTCTTCCGATCT-3′ to the MHC primer sequences (following Campbell et al. [71]). We performed PCRs in a total volume of 10 μl that contained; for MHC class I, 3.5 μl MM, 1.25 μl Q-solution, 1 μl of each primer (10 μM), 1 μl DNA (25 ng/μl) and 2.25 μl water, and for MHC class II, 4 μl MM, 1 μl of each primer (10 μM), 1 μl DNA (25 ng/μl) and 3 μl water. We used the same PCR programs as before but reduced the number of cycles to 28 to minimize the impact of chimera formation [72]. We then checked 1 μl of the products on an agarose gel and cleaned up the remainder of the solution using 8 μl (concentration of 0.8 X) of AMPure XP magnetic beads (Beckman Coulter, Indianapolis, USA) according to the manufacturer’s protocol. We suspended the clean product in 20 μl TE and transferred 4 μl of MHC class I and II amplicons to a new 96-well plate, combining the amplicons of both classes for the same individual for the PCR2. We then added 0.5 μl of 0.5 M forward and reverse Illumina indexes (dual-plexed Fi5 [12 indexes] and Ri7 [16 indexes]; index primers in the format 5′-[Illumina i5 or i7 capture sequence][6-bp i5 or i7 barcode] [overhang sequence]-3′, 10 μl MM and 1 μl water, and ran PCR2 using the following program: 95 °C for 15 min, followed by 9 cycles at 98 °C for 10 s, 66 °C for 30 s and 72 °C for 30 s, with a final step at 72 °C for 5 min.

We determined the concentration of the PCR2 product using a fluorometer (FLUOstar OPTIMA) and 2 μl of the product. We pooled samples from eight individuals by taking 20 ng per sample and cleaning up the multiplexed PCR products with AMPure XP beads, as described above, with volumes adjusted to a 50 μl solution (concentration of 0.5 X). We used a TapeStation 4200 (Agilent Genomics) to confirm that there were no primer dimers present in the purified samples. We then quantified the PCR products using a qPCR with the KAPA library quantification kit (KAPA Biosystem) using 10 μl reaction volume (8 μl of SYBR Master Mix and 2 μl of template/standard or control), with the program: 95 °C for 5 min, followed by 35 cycles of 94 °C for 30 s and 60 °C for 45 s. We then pooled equimolar amounts per library, preparing six libraries in total, quantified the concentration of the pool with a Qubit (ThermoFisher Scientific, Waltham, USA) and submitted 4 nM per library for sequencing using 250 bp paired-end (500 cycles) Illumina sequencing on the MiSeq (Illumina Inc., San Diego, CA, USA) in six separate runs at the Sheffield Diagnostic Genetics Service.

### Processing of data and MHC alleles validation

For the raw MiSeq data processing, we used the Amplicon Sequencing Analysis Tool (AmpliSAT) web server [73]. This tool is divided into different modules that allow the merging, cleaning and assignment of genotypes. First, we used AmpliMERGE with the FLASH algorithm to merge the pair-end reads. Then, we used AmpliCLEAN to filter out low-quality reads (<Q30 score and < 270 bp). After running AmpliCHECK with the default parameters for Illumina sequences, we retained all the remaining reads with lengths of 350 (325) ± 5 bp for MHC class I (II). Finally, we used AmpliSAS to demultiplex, cluster and filter the retained reads using default parameters for Illumina data for clustering, a minimum read depth per amplicon of 2000 and merging minimally different sequences to the dominant sequences when they differed by less than 3 bp and had ≤25% of the read depth in comparison with the dominant sequences [73]. Sequences that differed by 1–3 bp from the dominant sequences, but had more than 25% of the read depth, were classified as ‘subdominants’ and formed a new cluster representing a putative allele [26, 73]. We discarded all sequences that had a frequency of less than 5%, and those identified as chimera sequences. The minimum amplicon depth was set to 150 reads and the maximum amplicon depth set to 5000 reads due to computational limitations [73].

### Allele validation

We blasted all putative alleles from Illumina sequencing to the GenBank (NCBI) nucleotide database to examine their similarity to known MHC alleles from other species. The alleles were named Chni-UA*01 to UA*40 (MHC class I) and Chni-DAB*01 to DAB*06 (MHC class II), following the nomenclature suggested by Klein et al. [74].

### Diversity analysis and tests for selection

We used MEGA 7.0.18 [75] for initial diversity and selection analysis. First, we aligned the putative alleles using the MUSCLE algorithm [76] implemented in MEGA. We manually checked indel sites and curated alignments in order to preserve triplets within exons. We then estimated the number of segregating amino acid sites (*S*_aa_), nucleotide diversity (π), evolutionary distance for nucleotide sequences (*d*_nt_) and evolutionary distance for amino acid (*d*_aa_), for each of the eight populations included in the study. For the evolutionary distance analyses, we inferred the appropriate substitution models based on the best-fit model (using AIC_C_) using JModeltest 2.1.10 [77]. For MHC class I, we employed the Kimura two-parameter model [78] with a gamma distribution, setting the transition rate α to 0.9 for nucleotide sequences, and using the *p*-distance model with uniform rates to assess amino acid sequences distances. For MHC class II, we implemented the Jukes-Cantor + G model with a gamma distribution, setting the substitution rate α to 0.8 for nucleotide sequences, and we calculated amino acid sequence distances using a *p*-distance model with uniform rates.

We calculated standard errors of the mean evolutionary distances from 1000 bootstrap replicates. Positive selection, as a response to the selection imposed by pathogens, will lead to an excess of non-synonymous over synonymous substitutions in the PBR (ω = *dN*/*dS* > 1). To assess the impact of positive selection on nucleotide diversity, we compared ω at PBR sites and non-PBR sites. We inferred PBR sites based on previously documented transcripts (MHC class I: [79], MHC class II: [80]). We calculated synonymous and non-synonymous substitution rates through the modified Nei–Gojorobi method [81] with Jukes–Cantor correction. Also, we tested site-by-site selection applying Fast Unconstrained Bayesian AppRoximation (FUBAR; http://www.datamonkey.org/fubar, [82]. As recombination is frequent in MHC genes and recombination may lead to overestimation of the number of positively selected sites [62], we tested for evidence of recombination in our MHC alignments using Genetic Algorithm for Recombination Detection (GARD; http://www.datamonkey.org/gard, [83]).

### Phylogenetic diversity and relationships

We compared nucleotide diversity at MHC loci across the Charadriiformes using data available at GenBank. For MHC class I, we obtained sequences from three other charadriiform species: Red Knot *Calidris canutus* [25], Icelandic Black-tailed Godwit *Limosa limosa islandica* [26] and the Red-billed Gull *Larus scopulinus* [29]. As only sequences from 21 alleles were available for Red-billed Gull, we randomly drew sequences of 21 alleles from each species in this comparison to obtain a comparable sample size for the diversity estimate (Table S3). For MHC class II, we obtained data from seven further species: Common Murre *Uria alge* [44], Razorbill *Alca torda* [44], Atlantic Puffin *Fratercula artica* [44], Black-legged Kittiwake *Rissa tridactyla* [44], Great Snipe *Gallinago media* [28], Ruff *Philomachus pugnax* [16] and Black-tailed Godwit *Limosa limosa* [16]. As we had only six putative alleles in Snowy Plover, we capped the number of sequences to six per species that we randomly drew for this comparison (Table S3). We evaluated the amino acid (*S*_aa_) and nucleotide (*S*_nt_) segregation sites, nucleotide diversity (π), as well the synonymous (*dS*) and non-synonymous (*dN*) substitution rates, using the same parameters described above. We visualized phylogenetic relationships between the MHC class I and class II alleles in Snowy Plovers through the Neighbor-net algorithm implemented in SplitsTree 4.14.6 [84]. This method allows deduction of alternative phylogenetic histories and model incompatibilities in the dataset that may lead to conflicting phylogenetic signals because of duplication, recombination and gene conversion, which are all common in MHC genes [20, 25]. Finally, we inferred the phylogenetic relationships among charadriiform MHC alleles using a Neighbour-Joining Tree with Maximum Likelihood implemented in MEGA 7.0.18. We calculated branch support through 1000 bootstrap replications.

## Supplementary information

**Additional file 1: Figure S1.** Graphical representation of nucleotide diversity and d*N*/d*S* ratios for MHC class I and MHC class II. Silhouettes represent Charadriiform species, i.e. MHC class I (from top to bottom: Red Knot, Black-tailed Godwit, Red-billed Gull and Snowy Plover) and MHC class II (from top to bottom: Black-tailed Godwit, Ruff, Black-legged Kittiwake, Atlantic Puffin, Common Murre, Snowy Plover, Great Snipe and Razorbill). Arrows represent strength of selection in both MHC classes for the Snowy Plover in comparison to the other species. **Table S1.** Results of generalized linear models testing the differences in the number of alleles per individual between populations for the MHC class I and class II in the Snowy Plover. **Table S2.** Results of generalized linear models testing the differences in the number of alleles between populations for the MHC class I and class II in the Snowy Plover. **Table S3.** Sequences ID list randomly drawn for the MHC class I and MHC class II species comparison.

## Data Availability

All MHC allele sequences are available at GenBank, the sequence data base of the National Institutes of Health (NIH), USA. Accession numbers for MHC class I alleles are MT888135–MT888174 and for MHC class II alleles MT888175–MT888180.

## Abbreviations

AICc: Corrected Akaike information criterion
AmpliSAT: Amplicon Sequencing Analysis Tool
d*N*: Nonsynonymous substitution rate per nonsynonymous site
d*S*: Synonymous substitution rate per synonymous site
FUBAR: Fast Unconstrained Bayesian AppRoximation
GARD: Genetic Algorithm for Recombination Detection
MHC: Major histocompatibility complex
NCBI: National Center for Biotechnology Information
PBR: Peptide-binding region
PCR: Polymerase chain reaction
*S*_aa_: Amino acid segregation sites
*S*_nt_: Nucleotide segregation sites

## References

[1] Hess CM, Edwards SV (2002). The evolution of major histocompatibility genes in birds. Bioscience.

[2] Sommer S (2005). The importance of immune gene variability (MHC) in evolutionary ecology and conservation. Front Zool.

[3] Piertney SB, Oliver MK (2006). The evolutionary ecology of the major histocompatibility complex. Heredity.

[4] Spurgin LG, Richardson DS (2010). How pathogens drive genetic diversity: MHC, mechanisms and misunderstandings. Proc R Soc B.

[5] Bernatchez L, Landry C (2003). MHC studies in nonmodel vertebrates: what have we learned about natural selection in 15 years?. J Evol Biol.

[6] Eizaguirre C, Yeates SE, Lenz TL, Kalbe M, Milinski M (2009). MHC-based mate choice combines good genes and maintenance of MHC polymorphism. Mol Ecol.

[7] Siddle HV, Marzec J, Cheng Y, Jones M, Belov K (2010). MHC gene copy number variation in Tasmanian devils: implications for the spread of a contagious cancer. Proc R Soc B.

[8] Worley K, Collet J, Spurgin LG, Cornwallis C, Richardson DS (2010). MHC heterozygosity and survival in red junglefowl. Mol Ecol.

[9] Zagalska-Neubauer M, Babik W, Stuglik M, Gustafsson L, Cichoń M, Radwan J (2010). 454 sequencing reveals extreme complexity of the class II major histocompatibility complex in the collared flycatcher. BMC Evol Biol.

[10] Sepil I, Moghadam HK, Huchard E, Sheldon BC (2012). Characterization and 454 pyrosequencing of major histocompatibility complex class I genes in the great tit reveal complexity in a passerine system. BMC Evol Biol.

[11] Frankham R, Ballou JD, Briscoe DA (2010). Introduction to conservation genetics.

[12] Acevedo-Whitehouse K, Cunningham AA (2006). Is MHC enough for understanding wildlife immunogenetics?. Trends Ecol Evol.

[13] Edwards SV, Hedrick PW (1998). Evolution and ecology of MHC molecules: from genomics to sexual selection. Trends Ecol Evol.

[14] Babik W (2010). Methods for MHC genotyping in non-model vertebrates. Mol Ecol Resour.

[15] Oosterhout VP (2009). A new theory of MHC evolution: beyond selection on the immune genes. Proc R Soc B.

[16] Burri R, Promerov M, Goebel J, Fumagalli L (2014). PCR-based isolation of multigene families: lessons from the avian MHC class IIB. Mol Ecol Resour.

[17] Minias P, Pikus E, Whittingham LA, Dunn PO (2018). Evolution of copy number at the MHC varies across the avian tree of life. Genome Biol Evol.

[18] Winternitz J, Abbate JL, Huchard E, Havlicek J, Garamszegi LZ (2017). Patterns of MHC-dependent mate selection in humans and nonhuman primates: a meta-analysis. Mol Ecol.

[19] Kaufman J, Milne S, Göbel T, Walker BA, Jacob JP, Auffrey C (1999). The chicken B locus is a minimal-essential major histocompatibility complex. Nature.

[20] Alcaide M, Edwards SV, Cadahía L, Negro JJ (2009). MHC class I genes of birds of prey: isolation, polymorphism and diversifying selection. Conserv Genet.

[21] Shiina T, Shimizu S, Hosomichi K, Kohara S, Watanabe S, Hanzawa K (2004). Comparative genomic analysis of two avian (quail and chicken) MHC regions. J Immunol.

[22] Chaves LD, Krueth SB, Reed KM (2009). Defining the Turkey MHC: sequence and genes of the B locus. J Immunol.

[23] Westerdahl H, Wittzell H, von Schantz T (1999). Polymorphism and transcription of Mhc class I genes in a passerine bird, the great reed warbler. Immunogenetics.

[24] Karlsson M, Westerdahl H (2013). Characteristics of MHC class I genes in house sparrows *Passer domesticus* as revealed by long cDNA transcripts and amplicon sequencing. J Mol Evol.

[25] Buehler DM, Verkuil YI, Tavares ES, Baker AJ (2013). Characterization of MHC class I in a long-distance migrant shorebird suggests multiple transcribed genes and intergenic recombination. Immunogenetics.

[26] Pardal S, Drews A, Alves JA, Ramos JA, Westerdahl H (2017). Characterization of MHC class I in a long distance migratory wader, the Icelandic black-tailed godwit. Immunogenetics.

[27] Nei M, Gu X, Sitnikova T (1997). Evolution by the birth-and-death process in multigene families of the vertebrate immune system. Proc Natl Acad Sci.

[28] Ekblom R, Sæther SA, Jacobsson P, Fiske P, Sahlman T, Grahn M (2007). Spatial pattern of MHC class II variation in the great snipe (*Gallinago media*). Mol Ecol.

[29] Cloutier A, Mills J, Baker A (2011). Characterization and locus-specific typing of MHC class I genes in the red-billed gull (*Larus scopulinus*) provides evidence for major, minor, and nonclassical loci. Immunogenetics.

[30] dos Remedios N, Lee PLM, Burke T, Székely T, Küpper C (2015). North or south? Phylogenetic and biogeographic origins of a globally distributed avian clade. Mol Phylogenet Evol.

[31] Funk WC, Mullins TD, Haig SM (2007). Conservation genetics of Snowy Plovers (*Charadrius alexandrinus*) in the western hemisphere: population genetic structure and delineation of subspecies. Conserv Genet.

[32] Küpper C, Augustin J, Kosztolányi A, Burke T, Figuerola J, Székely T (2009). Kentish versus Snowy Plover: phenotypic and genetic analyses of *Charadrius alexandrinus* reveal divergence of Eurasian and American subspecies. Auk.

[33] D’Urban Jackson J, Bruford MW, Székely T, DaCosta JM, Sorenson MD, Edwards SV (2020). Population differentiation and historical demography of the threatened Snowy Plover *Charadrius nivosus* (Cassin, 1858). Conserv Genet.

[34] Küpper C, Aguilar E, Gonzalez O (2011). Notas sobre la ecología reproductiva y conservación de los chorlos nevados *Charadrius nivosus occidentalis* en Paracas, Perú. Rev Peru Biol.

[35] Thomas SM, Lyons JE, Andres BA, T-Smith EE, Palacios E, Cavitt JF (2012). Population size of Snowy Plovers breeding in North America. Waterbirds.

[36] Galindo-Espinosa D, Palacios E (2015). Estatus del chorlo nevado (*Charadrius nivosus*) en San Quintín y su disminución poblacional en la península de Baja California. Rev Mex Biodivers.

[37] Cruz-López M, Eberhart-Phillips LJ, Fernández G, Beamonte-Barrientos R, Székely T, Serrano-Meneses MA (2017). The plight of a plover: viability of an important Snowy Plover population with flexible brood care in Mexico. Biol Conserv.

[38] Saper MA, Bjorkman PJ, Wiley DC (1991). Refined structure of the human histocompatibility antigen HLA-A2 at 2.6 Å resolution. J Mol Biol.

[39] Rodgers JR, Cook RG (2005). MHC class Ib molecules bridge innate and acquired immunity. Nat Rev Immunol.

[40] Krovi SH, Gapin L (2016). Structure and function of the non-classical major histocompatibility complex molecule MR1. Immunogenetics.

[41] Hosomichi K, Miller MM, Goto RM, Wang Y, Suzuki S, Kulski JK, Nishibori M, Inoko H, Hanzawa K, Shiina T (2008). Contribution of mutation, recombination, and gene conversion to chicken MHC-B haplotype diversity. J Immunol.

[42] Minias P, Pikus E, Whittingham LA, Dunn PO (2018). A global analysis of selection at the avian MHC. Evolution.

[43] Tian D, Wang Q, Zhang P, Araki H, Yang S, Kreitman M (2008). Single-nucleotide mutation rate increases close to insertions/deletions in eukaryotes. Nature.

[44] Sequence variation in the MHC class II DRB1-like gene of four colonial seabirds. GenBank. 2010. Available from: https://www.ncbi.nlm.nih.gov. Cited 15 May 2019.

[45] Poulin R (1995). Phylogeny, ecology, and the richness of parasite communities in vertebrates. Ecol Monogr.

[46] Page GW, Stenzel LE, Warriner JS, Warriner JC, Paton PW (2009). Snowy Plover (*Charadrius nivosus*), version 2.0. The birds of North America.

[47] Figuerola J (1999). Effects of salinity on rates of infestation of waterbirds by haematozoa. Ecography.

[48] Mendes L, Piersma T, Lecoq M, Spaans B, Ricklefs RE (2005). Disease-limited distributions? Contrasts in the prevalence of avian malaria in shorebird species using marine and freshwater habitats. Oikos.

[49] Poulin R (2016). Greater diversification of freshwater than marine parasites of fish. Int J Parasitol.

[50] McLean RG, Ubico SR, Thomas NJ, Hunter DB, Atkinson CT (2007). Arboviruses in birds. Infectious diseases of wild birds.

[51] Iverson SA, Takekawa JY, Schwarzbach S, Gardona CJ, Warnock N, Bishop MA (2008). Low prevalence of avian influenza virus in shorebirds on the Pacific coast of North America. Waterbirds.

[52] Clark NJ, Clegg SM, Klaassen M (2016). Migration strategy and pathogen risk: non-breeding distribution drives malaria prevalence in migratory waders. Oikos.

[53] Santos SS, Pardal S, Proença DN, Lopes RJ, Ramos JA, Mendes L (2012). Diversity of cloacal microbial community in migratory shorebirds that use the Tagus estuary as stopover habitat and their potential to harbor and disperse pathogenic microorganisms. FEMS Microbiol Ecol.

[54] D’Urban Jackson J, dos Remedios N, Maher KH, Zefania S, Haig S, Oyler-Mccance S (2017). Polygamy slows down population divergence in shorebirds. Evolution.

[55] Morand S, Bordes F, Pisanu B, Goüy de Bellocq J, Krasnov BR, Morand S, Krasnov BR (2010). The geography of defence. The biogeography of host-parasite interactions.

[56] Rohde K, Morand S, Krasnov BR (2010). Marine parasite diversity and environmental gradients. The biogeography of host–parasite interactions.

[57] Eimes JA, Reed KM, Mendoza KM, Bollmer JL, Whittingham LA, Bateson ZW (2013). Greater prairie chickens have a compact MHC-B with a single class IA locus. Immunogenetics.

[58] Minias P, Bateson Z, Whittingham LA, Johnson JA, Oyler-McCance S, Dunn PO (2016). Contrasting evolutionary histories of MHC class I and class II in grouse–effects of selection and gene conversion. Heredity.

[59] Minias P, Pikus E, Anderwald D (2019). Allelic diversity and selection at the MHC class I and class II in a bottlenecked bird of prey, the White-tailed Eagle. BMC Evol Biol.

[60] Vásquez-Carrillo C, Friesen V, Hall L, Peery MZ (2014). Variation in MHC class II B genes in marbled murrelets: implications for delineating conservation units. Anim Conserv.

[61] Sallaberry-Pincheira N, González-Acuña D, Padilla P, Dantas GPM, Luna-Jorquera G, Frere E (2016). Contrasting patterns of selection between MHC I and II across populations of Humboldt and Magellanic penguins. Ecol Evol.

[62] Alcaide M, Muñoz J, Martínez-de la Puente J, Soriguer R, Figuerola J (2014). Extraordinary MHC class II B diversity in a non-passerine, wild bird: the Eurasian Coot *Fulica atra* (Aves: Rallidae). Ecol Evol.

[63] Strand T, Westerdahl H, Höglund J, Alatalo RV, Siitari H (2007). The Mhc class II of the Black grouse (*Tetrao tetrix*) consists of low numbers of B and Y genes with variable diversity and expression. Immunogenetics.

[64] Székely T, Kosztolanyi A, Küpper C. Practical guide for investigating breeding ecology of Kentish Plover (*Charadrius alexandrinus*): University of Bath; 2008. Unpublished report. http://www.pennuti.net/wp-content/uploads/2010/08/KP_Field_Guide_v3.pdf.

[65] Seutin G, White BN, Boag PT (1991). Preservation of avian blood and tissue samples for DNA analyses. Can J Zool.

[66] Nicholls JA, Double MC, Rowell DM, Magrath RD (2000). The evolution of cooperative and pair breeding in thornbills *Acanthiza* (Pardalotidae). J Avian Biol.

[67] Alcaide M, Cadahía L, Lambertucci SA, Negro JJ (2010). Noninvasive estimation of minimum population sizes and variability of the major histocompatibility complex in the Andean Condor. Condor.

[68] Miller HC, Lambert DM (2004). Gene duplication and gene conversion in class II MHC genes of New Zealand robins (Petroicidae). Immunogenetics.

[69] Edwards SV, Grahn M, Potts WK (1995). Dynamics of MHC evolution in birds and crocodilians: amplification of class II genes with degenerate primers. Mol Ecol.

[70] Alcaide M, Edwards SV, Negro JJ (2007). Characterization, polymorphism, and evolution of MHC class II B genes in birds of prey. J Mol Evol.

[71] Campbell NR, Harmon SA, Narum SR (2015). Genotyping-in-thousands by sequencing (GT-seq): a cost effective SNP genotyping method based on custom amplicon sequencing. Mol Ecol Resour.

[72] Lenz TL, Becker S (2008). Simple approach to reduce PCR artefact formation leads to reliable genotyping of MHC and other highly polymorphic loci —implications for evolutionary analysis. Gene.

[73] Sebastian A, Herdegen M, Migalska M, Radwan J (2016). Amplisas: a web server for multilocus genotyping using next-generation amplicon sequencing data. Mol Ecol Resour.

[74] Klein J, Bontrop RE, Dawkins RL, Erlich HA, Gyllensten UB, Heise ER (1990). Nomenclature for the major histocompatibility complexes of different species: a proposal. Immunogenetics.

[75] Kumar S, Stecher G, Tamura K (2016). MEGA7: molecular evolutionary genetics analysis version 7.0 for bigger datasets. Mol Biol Evol.

[76] Edgar RC (2004). Muscle: a multiple sequence alignment method with reduced time and space complexity. BMC Bioinformatics.

[77] Darriba D, Taboada GL, Doallo R, Posada D (2012). jModelTest 2: more models, new heuristics and high-performance computing. Nat Methods.

[78] Kimura M (1980). A simple method for estimating evolutionary rates of base substitutions through comparative studies of nucleotide sequences. J Mol Evol.

[79] Wallny HJ, Avila D, Hunt LG, Powell TJ, Riegert P, Salomonsen J (2006). Peptide motifs of the single dominantly expressed class I molecule explain the striking MHC-determined response to *Rous sarcoma* virus in chickens. Proc Natl Acad Sci.

[80] Brown JH, Jardetzky T, Saper MA, Samraoui B, Bjorkman PJ, Wiley DC (1988). A hypothetical model of the foreign antigen binding site of class II histocompatibility molecules. Nature.

[81] Nei M, Gojobori T (1986). Simple methods for estimating the numbers of synonymous and nonsynonymous nucleotide substitutions. Mol Biol Evol.

[82] Murrell B, Moola S, Mabona A, Weighill T, Sheward D, Pond SLK (2013). FUBAR: a fast, unconstrained Bayesian AppRoximation for inferring selection. Mol Biol Evol.

[83] Pond SLK, Posada D, Gravenor MB, Woelk CH, Frost SD (2006). Automated phylogenetic detection of recombination using a genetic algorithm. Mol Biol Evol.

[84] Huson DH, Bryant D (2006). Application of phylogenetic networks in evolutionary studies. Mol Biol Evol.

