# Supplementary material for: Allelic diversity and patterns of selection at the major histocompatibility complex class I and II loci in a threatened shorebird, the Snowy Plover (Charadrius nivosus)
Source: BMC Evol Biol. 2020 Sep 10;20:114. doi: 10.1186/s12862-020-01676-7 (PMC7488298; doi:10.1186/s12862-020-01676-7)
Supplement: Supplementary file 1 — Additional file 1: Figure S1. Graphical representation of nucleotide diversity and dN/dS ratios for MHC class I and MHC class II. Silhouettes represent Charadriiform species, i.e. MHC class I (from top to bottom: Red Knot, Black-tailed Godwit, Red-billed Gull and Snowy Plover) and MHC class II (from top to bottom: Black-tailed Godwit, Ruff, Black-legged Kittiwake, Atlantic Puffin, Common Murre, Snowy Plover, Great Snipe and Razorbill). Arrows represent strength of selection in both MHC classes for the Snowy Plover in comparison to the other species. Table S1. Results of generalized linear models testing the differences in the number of alleles per individual between populations for the MHC class I and class II in the Snowy Plover. Table S2. Results of generalized linear models testing the differences in the number of alleles between populations for the MHC class I and class II in the Snowy Plover. Table S3. Sequences ID list randomly drawn for the MHC class I and MHC class II species comparison. [file 12862_2020_1676_MOESM1_ESM.docx]

**Additional file 1.**

Figure S1. Graphical representation of nucleotide diversity and dN/dS ratios for MHC class I and MHC class II. Silhouettes represent Charadriiform species, i.e. MHC class I (from top to bottom: Red Knot, Black-tailed Godwit, Red-billed Gull and Snowy Plover) and MHC class II (from top to bottom: Black-tailed Godwit, Ruff, Black-legged Kittiwake, Atlantic Puffin, Common Murre, Snowy Plover, Great Snipe and Razorbill). Arrows represent strength of selection in both MHC classes for the Snowy Plover in comparison to the other species.


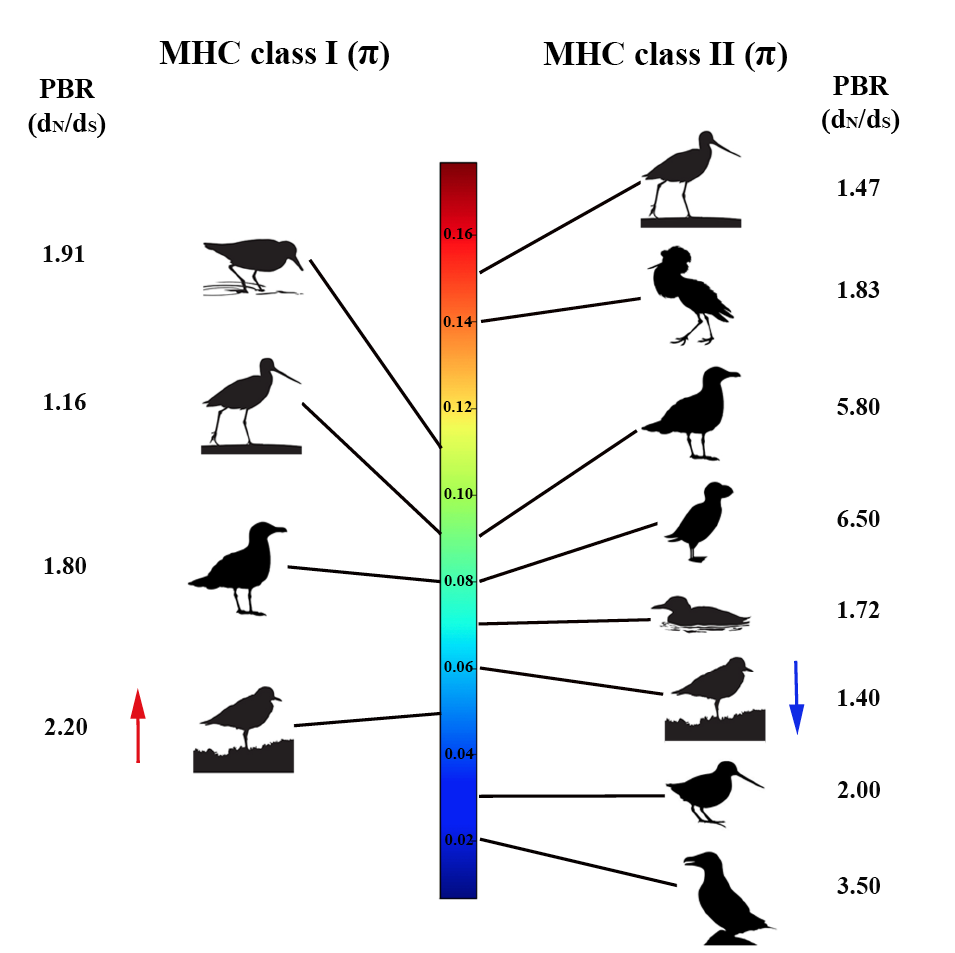


Table S1. Results of generalized linear models testing the differences in the number of alleles per individual between populations for the MHC class I and class II in the Snowy Plover.

Table S2. Results of generalized linear models testing the differences in the number of alleles between populations for the MHC class I and class II in the Snowy Plover.

Table S3. Sequences ID list randomly drawn for the MHC class I and MHC class II species comparison.

| Exon 3 MHC I Charadriiformes sequences ID | | Exon 2 MHC II Charadriiformes sequences ID | |
| --- | --- | --- | --- |
| Species ID | Sequences ID | Species ID | Sequences ID |
| \| Calidris_canutus \| \| --- \| \| Calidris_canutus \| \| Calidris_canutus \| \| Calidris_canutus \| \| Calidris_canutus \| \| Calidris_canutus \| \| Calidris_canutus \| \| Calidris_canutus \| \| Calidris_canutus \| \| Calidris_canutus \| \| Calidris_canutus \| \| Calidris_canutus \| \| Calidris_canutus \| \| Calidris_canutus \| \| Calidris_canutus \| \| Calidris_canutus \| \| Calidris_canutus \| \| Calidris_canutus \| \| Calidris_canutus \| \| Calidris_canutus \| \| Calidris_canutus \| \| Larus_scopulinus \| \| Larus_scopulinus \| \| Larus_scopulinus \| \| Larus_scopulinus \| \| Larus_scopulinus \| \| Larus_scopulinus \| \| Larus_scopulinus \| \| Larus_scopulinus \| \| Larus_scopulinus \| \| Larus_scopulinus \| \| Larus_scopulinus \| \| Larus_scopulinus \| \| Larus_scopulinus \| \| Larus_scopulinus \| \| Larus_scopulinus \| \| Larus_scopulinus \| \| Larus_scopulinus \| \| Larus_scopulinus \| \| Larus_scopulinus \| \| Larus_scopulinus \| \| Larus_scopulinus \| \| Limosa_limosa \| \| Limosa_limosa \| \| Limosa_limosa \| \| Limosa_limosa \| \| Limosa_limosa \| \| Limosa_limosa \| \| Limosa_limosa \| \| Limosa_limosa \| \| Limosa_limosa \| \| Limosa_limosa \| \| Limosa_limosa \| \| Limosa_limosa \| \| Limosa_limosa \| \| Limosa_limosa \| \| Limosa_limosa \| \| Limosa_limosa \| \| Limosa_limosa \| \| Limosa_limosa \| \| Limosa_limosa \| \| Limosa_limosa \| \| Limosa_limosa \| | \| KC205106.1 \| \| --- \| \| KC205107.1 \| \| KC205109.1 \| \| KC205111.1 \| \| KC205112.1 \| \| KC205113.1 \| \| KC205114.1 \| \| KC205115.1 \| \| KC205117.1 \| \| KC205118.1 \| \| KC205119.1 \| \| KC205121.1 \| \| KC205122.1 \| \| KC205123.1 \| \| KC205124.1 \| \| KC205125.1 \| \| KC205126.1 \| \| KC205127.1 \| \| KC205128.1 \| \| KC205129.1 \| \| KC205130.1 \| \| HM025950.1 \| \| HM025953.1 \| \| HM025954.1 \| \| HM025955.1 \| \| HM025956.1 \| \| HM025957.1 \| \| HM025958.1 \| \| HM025959.1 \| \| HM025960.1 \| \| HM025961.1 \| \| HM025962.1 \| \| HM025963.1 \| \| HM025964.1 \| \| HM025965.1 \| \| HM025966.1 \| \| HM025968.1 \| \| HM025969.1 \| \| HM025970.1 \| \| HM025971.1 \| \| HM025972.1 \| \| HM025974.1 \| \| KY351552.1 \| \| KY351553.1 \| \| KY351554.1 \| \| KY351555.1 \| \| KY351559.1 \| \| KY351560.1 \| \| KY351561.1 \| \| KY351564.1 \| \| KY351565.1 \| \| KY351567.1 \| \| KY351569.1 \| \| KY351571.1 \| \| KY351572.1 \| \| KY351573.1 \| \| KY351576.1 \| \| KY351577.1 \| \| KY351578.1 \| \| KY351579.1 \| \| KY351580.1 \| \| KY351583.1 \| \| KY351585.1 \| | \| Alca_torda \| \| --- \| \| Alca_torda \| \| Alca_torda \| \| Alca_torda \| \| Alca_torda \| \| Alca_torda \| \| Fratercula_arctica \| \| Fratercula_arctica \| \| Fratercula_arctica \| \| Fratercula_arctica \| \| Fratercula_arctica \| \| Fratercula_arctica \| \| Gallinago_media \| \| Gallinago_media \| \| Gallinago_media \| \| Gallinago_media \| \| Gallinago_media \| \| Gallinago_media \| \| Limosa_limosa \| \| Limosa_limosa \| \| Limosa_limosa \| \| Limosa_limosa \| \| Philomachus_pugnax \| \| Philomachus_pugnax \| \| Philomachus_pugnax \| \| Philomachus_pugnax \| \| Philomachus_pugnax \| \| Philomachus_pugnax \| \| Rissa_tridactyla \| \| Rissa_tridactyla \| \| Rissa_tridactyla \| \| Rissa_tridactyla \| \| Rissa_tridactyla \| \| Rissa_tridactyla \| \| Uria_aalge \| \| Uria_aalge \| \| Uria_aalge \| \| Uria_aalge \| \| Uria_aalge \| \| Uria_aalge \| | \| HQ822514.1 \| \| --- \| \| HQ822520.1 \| \| HQ822524.1 \| \| HQ822528.1 \| \| HQ822521.1 \| \| EU326271.1 \| \| HQ822503.1 \| \| HQ822509.1 \| \| HQ822500.1 \| \| HQ822494.1 \| \| HQ822491.1 \| \| HQ822485.1 \| \| AF485417.1 \| \| AF485414.1 \| \| AY694409.1 \| \| AY694405.1 \| \| AY694385.1 \| \| AY620012.1 \| \| KJ162434.1 \| \| KJ162435.1 \| \| KJ162436.1 \| \| KJ162437.1 \| \| KJ162443.1 \| \| KJ162446.1 \| \| KJ162447.1 \| \| KJ162448.1 \| \| XM014959847 \| \| XM014959856 \| \| EU326257.1 \| \| EU326244.1 \| \| HQ822471.1 \| \| HQ822448.1 \| \| HQ822408.1 \| \| HQ822398.1 \| \| HQ822481.1 \| \| HQ822480.1 \| \| HQ822479.1 \| \| HQ822475.1 \| \| HQ822474.1 \| \| HQ822477.1 \| |
